# A Comparative Analysis of EWT and EMD Techniques in the Diagnosis of Major Depressive Disorder Using EEG: Asymmetry Features and Explainability via SHAP

**DOI:** 10.3390/diagnostics16132107

**Published:** 2026-07-05

**Authors:** Nadide Gulsah Gulenc, Gokce Koc, Mahmut Ozturk

**Affiliations:** 1 Department of Biomedical Engineering, Çorlu Engineering Faculty, Tekirdag Namık Kemal University, Tekirdag 59860, Türkiye; ngulsahgulenc@nku.edu.tr; 2 Department of Biomedical Engineering, Institute of Graduate Studies, Istanbul University-Cerrahpasa, Istanbul 34320, Türkiye; 3 Biomedical Engineering Department, Faculty of Engineering and Architecture, Istanbul Yeni Yuzyil University, Istanbul 34010, Türkiye; 4 Department of Electrical and Electronics Engineering, Engineering Faculty, Istanbul University-Cerrahpasa, Istanbul 34320, Türkiye; mahmutoz@iuc.edu.tr

**Keywords:** major depressive disorder, EEG, EMD, EWT, machine learning, SHAP, asymmetry features

## Abstract

**Background/Objectives**: Major Depressive Disorder is a serious mental disorder that negatively affects an individual’s health and quality of life. The diagnosis of this disease is based on clinical interviews, questionnaires, and the patient’s self-reports. The objective of this study is to develop a biological diagnostic system based on the analysis of EEG signals and brain regions, rather than relying on self-reports. **Methods**: In this study, the EEG signals in the Multimodal Open Mental Disorder Analysis (MODMA) dataset were divided into six anatomical regions: prefrontal, frontal, central, parietal, temporal, and occipital. Empirical Wavelet Transform and Empirical Mode Decomposition methods were applied separately to the channels in each region, resulting in three IMF components. A total of 23 features, including statistical, nonlinear, spectral, and model-based (AR) features, were extracted from each IMF component. In addition to these features, asymmetry features between the left and right hemispheres were also included. Feature dimensions ranging from 10 to 40 were selected via the mRMR method, and the extracted feature sets were classified using SVM, k-NN, RUSBoost, Random Forest, and Meta-Ensemble machine learning models with Leave-One-Subject-Out (LOSO) validation. **Results**: According to the analysis results, the highest accuracy rate in Major Depressive Disorder (MDD) diagnosis was achieved by classifying features extracted from the frontal and prefrontal regions. The EMD signal processing method demonstrated superior performance compared to the EWT method. An accuracy rate of 98.11% was achieved using Random Forest and Meta-Ensemble models. **Conclusions**: In the proposed method, Explainable Artificial Intelligence (XAI) based SHAP analysis was applied to provide reliable and interpretable features for MDD diagnosis based on brain regional analysis.

## 1. Introduction

Depression is defined as a psychiatric disorder that affects an individual’s mood, thought patterns, and daily functioning. It is not merely a temporary state of sadness; rather, it represents a more complex clinical condition characterized by symptoms such as loss of interest, decreased motivation, low energy, and a decline in cognitive performance. Major Depressive Disorder (MDD) is a more severe and clinically significant form of depression, marked by persistent mood disturbances, loss of interest, and reduced cognitive functioning [1,2].

Currently, MDD is typically diagnosed through clinical interviews and standard assessment scales (HDRS, BDI, DRS, ICD-10, MINI, PHQ-9). However, since these approaches rely largely on subjective reports and clinical interpretation, the diagnostic process may show some variability. In addition, they do not directly reflect the underlying neurophysiological mechanisms of depression. Therefore, there is a growing need for more objective and measurable diagnostic approaches based on biological markers [3].

The increasing prevalence of depression has led to its recognition as a major global public health concern. As a result, early and accurate detection has become increasingly important. In this context, research focusing on objective and measurable methods for depression assessment has gained significant attention. Among these methods, electroencephalography (EEG) has emerged as a prominent tool due to its non-invasive nature, low cost, portability, and high temporal resolution [4]. EEG enables the investigation of dynamic neurophysiological processes by directly measuring brain activity through electrical signals [5]. This makes it valuable not only for diagnosis but also for monitoring treatment and predicting treatment response. Previous studies have shown that changes in specific frequency components of EEG signals are associated with antidepressant treatments and neuromodulation methods [4].

In the literature, various feature extraction and classification approaches have been applied to EEG-based depression detection. However, in some studies, the frequency content of EEG signals is represented in a limited manner, and feature extraction methods are generally applied at a single level [6,7]. In addition, many studies analyze EEG signals across all channels collectively, while analyses based on brain regions are considered only to a limited extent. It has also been observed that nonlinear features and feature selection strategies are not always explored in detail. As a result, high-dimensional feature sets may be directly used in modeling, which can affect classification performance [3,8].

To address these limitations, this study proposes an integrated approach. EEG signals are first grouped into anatomically meaningful brain regions (prefrontal, frontal, central, parietal, temporal, and occipital) to enable analysis based on brain regions. EWT and EMD are then applied separately to each region to decompose the signals into sub-components. From these components, statistical, spectral, Hjorth, entropy, energy, and model-based (AR) features are extracted. In addition, asymmetry features are included to capture interhemispheric differences. Finally, the mRMR method is used to select the most informative features from the extracted feature set.

The selected features are evaluated using SVM, k-NN, Random Forest, RUSBoost, and Meta-Ensemble classifiers. Model performance is assessed using the Leave-One-Subject-Out (LOSO) cross-validation method. In addition, SHAP-based analysis is employed to improve model interpretability.

The main contributions of this study can be summarized as follows:

- Analysis of EEG signals based on brain regions
- Use of a multilayer decomposition approach based on EWT and EMD
- Combined use of statistical, spectral, and nonlinear features
- Application of mRMR-based feature selection
- Comparative evaluation of multiple machine learning models
- Use of SHAP for model interpretability

The proposed approach differs from existing methods by integrating analysis based on brain regions with the multi-scale structure of EEG signals, providing a more comprehensive framework for depression detection.

The remainder of this article is organized as follows: Section 2 (Related Studies) presents a comprehensive literature review of studies utilizing the MODMA dataset for EEG-based depression detection, highlighting their achievements and methodological approaches. Section 3 (Methodology) details the EEG signal preprocessing, the theoretical foundations of the EWT and EMD methods, feature extraction and selection processes, and machine learning architectures. Section 4 (Results and Discussion) presents statistical significance tests using XAI-based SHAP analysis, as well as comparative performance analyses of classification models across different brain regions. Furthermore, it discusses the study’s limitations and the main findings that could provide a foundation for future research on the detection of major depressive disorder. Finally, in Section 5 (Conclusions), the study’s main conclusions are summarized based on the analyses, promising biomarkers for the detection of major depressive disorder are discussed, and recommendations for future research are presented.

## 2. Related Works

The MODMA dataset is one of the open-access datasets widely used in the literature for EEG-based depression analysis. It offers an important advantage by providing both 128-channel high-resolution EEG recordings and three-channel EEG data suitable for portable systems [9].

In studies conducted using this dataset, various channel selections, feature extraction methods, and classification algorithms have been proposed. In recent years, the use of both machine learning and deep learning-based approaches has become increasingly widespread. A comparative summary of these studies is presented in Table 1.

When examining Table 1, it is seen that most of the existing studies either analyze raw EEG signals directly or use a limited number of feature extraction methods. Studies that consider multilayer signal decomposition together with nonlinear features remain limited. Despite the existing studies, it is emphasized in various sources that the number of studies on the use of EEG signals in the diagnosis of MDD is still limited, and more scientific studies are needed in this field [19].

In addition, although some studies report high accuracy rates, it is observed that these results are often obtained using methods such as high model complexity, deep learning architectures, or multiple data fusion approaches. While such approaches offer strong results in terms of performance, they may have certain limitations in terms of interpretability and generalizability [8,10].

In this study, an approach combining adaptive signal decomposition methods with machine learning for the diagnosis of major depressive disorder (MDD) using EEG signals is proposed. Signals obtained from the MODMA dataset were first segmented according to brain regions and preprocessed, and then decomposed into their subcomponents using the EWT and EMD methods. Features derived from these components were selected using the mRMR method, and classification was performed using various machine learning algorithms. Model performance was evaluated using the Leave-One-Subject-Out (LOSO) cross-validation method, and the decision mechanism was examined via SHAP analysis.

## 3. Methodology

**Figure 1:**
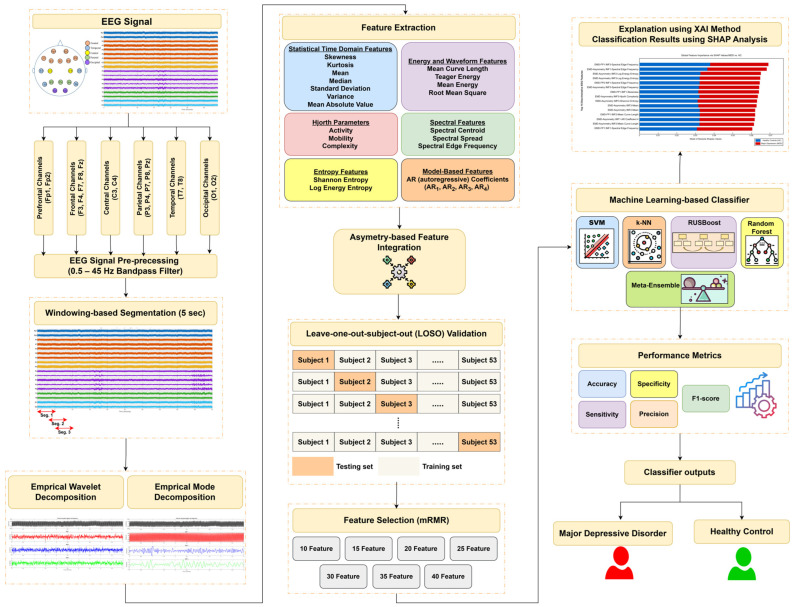
Flowchart of the proposed study.

This section presents the flowchart and stages of the proposed method for diagnosing MDD using EEG signals, as shown in Figure 1. The proposed method consists of the following stages: preprocessing, signal decomposition into sub-bands, feature extraction, feature selection, and classification.

In the first stage, the channels in the EEG signals from the MODMA dataset were separated according to the prefrontal, frontal, central, parietal, temporal, and occipital brain regions. Bandpass filters with a frequency range of 0.5–45 Hz were applied to remove noise and artifacts from these signals. The filtered EEG signals were divided into 5-s segments with a 2.5-s overlap. Each channel was decomposed into three sub-bands using both the Empirical Wavelet Transform and Empirical Mode Decomposition signal processing methods to further analyze the structure of the complex and non-stationary EEG signals. Twenty-three features—including statistical, spectral, Hjorth, entropy, energy, and model-based (AR) features—were extracted from these sub-bands to represent the physiological characteristics of the EEG signal. In addition to these features, asymmetry features between channels were also integrated into the analysis process to characterize interhemispheric relationships. The Leave-One-Subject-Out (LOSO) validation method was utilized to evaluate the performance of the classification models. To prevent data leakage, all data-dependent preprocessing steps, including mRMR feature selection, were performed strictly within each LOSO training fold. Specifically, in each iteration, all data belonging to a single subject were completely set aside as the test set. The data from the remaining N − 1 subjects were used exclusively as the training set, upon which the mRMR method was applied to identify the most discriminative features. Within this framework, various feature sets containing between 10 and 40 features were prepared and analyzed for each fold. As a result, the test data was completely separated throughout the feature selection and training phases. This allowed for an assessment of the model’s generalizability to new individuals. In this study, SVM, RUSBoost, k-NN, Random Forest, and Meta-Ensemble were used as classification methods. In the final stage, the models’ performance was assessed using performance metrics such as accuracy, precision, recall, and F1 score. In recent analyses of EEG signals, it is recommended that these performance metrics be used to evaluate the classification performance of machine learning models in an unbiased and objective manner [20,21]. The models were analyzed using SHAP (SHapley Additive exPlanations) to determine which features were more effective in classification and interpret the model’s decision-making mechanism. Therefore, the classification results were supported by integrating explainable artificial intelligence (XAI) in this study. The overall workflow of the proposed framework is shown in Figure 1.

### 3.1. Dataset

The Multimodal Open Mental Disorder Analysis (MODMA) dataset, created in 2020 by the Second Hospital of Lanzhou University in China, was used in this study. The dataset consists of EEG signals and audio recordings from healthy individuals and individuals diagnosed with depression, collected from a 128-channel resting-state EEG device and a 3-channel wearable device. In this study, 128-channel EEG signals from 53 participants in a resting state were used, including 24 with MDD (aged 16–56; 13 men and 11 women) and 29 healthy individuals (aged 18–55; 20 men and 9 women). The signals were sampled at a frequency of 250 Hz. Additionally, psychiatrists assessed all participants’ general health, psychosocial status, sleep quality, and other relevant factors through a questionnaire. The MDD diagnosis for participants in the dataset was made by a specialist psychiatrist based on the DSM-IV criteria in effect at the time of the study. Psychiatrists also clinically verified the diagnoses through the Mini International Neuropsychiatric Interview (MINI) and confirmed them using Patient Health Questionnaire-9 (PHQ-9) scores. The PHQ-9 was used to define a score of 5 or higher as MDD and a score below 5 as the healthy control group [9].

### 3.2. Preprocessing of the EEG Signals

EEG signals, which provide information about the brain’s electrical activity and a person’s neurological condition, were filtered to remove noise components using appropriate preprocessing methods. As Chen et al. highlighted, noise removal and preprocessing of EEG signals are of critical importance for reliable feature evaluation and successful classification [22]. In this study, a bandpass filter was applied to all channels in the cortical region of the study to eliminate noise in the EEG signals. A bandpass filter was applied in the 0.5–45 Hz frequency band to eliminate low-frequency drifts, environmental noise, and electrical line noise. In studies on EEG signals in the literature, this bandpass filter was generally applied to remove noise for the detection of neurological conditions.

A fourth-order Butterworth bandpass filter and a forward-backward filtering (filtfilt) algorithm were applied to the signals to preserve peak values and phase information in the time domain. Thus, unwanted frequency components and phase distortions in the signals were minimized. Characteristic features for MDD diagnosis were maintained by applying preprocessing [23,24]. These analyses were applied on a laptop equipped with an Intel(R) Core(TM) i7-8550U processor operating at 1.80 GHz, a 64-bit operating system, and 8 GB of RAM. All signal processing, feature extraction, and classification operations were carried out using MATLAB 2025b.

### 3.3. Empirical Wavelet Transform (EWT)

Empirical Wavelet Transform signal processing method was proposed by J. Gilles in 2013 for the analysis of non-stationary signals [25]. The EWT method has a wavelet filter bank structure and decomposes the signal into appropriate modes in the Fourier spectrum. The advantage of the EWT method is that it automatically determines the spectral boundaries based on the signal’s non-stationary nature. The following steps are applied to an original f(t) signal for the EWT method [26,27].

Step 1: The Fourier spectrum of the f(t) signal is obtained. Local maxima are identified on this spectrum. The spectrum is divided into N segments in the interval [0, π]. The boundaries ω_n_ for the segmentation of the spectrum are calculated as the midpoints between two local maxima, as shown in Equation (1). $W_{i}$ and $W_{i+1}$ are two consecutive frequency maximum points [26,27].

(1) $$\omega_{i}=\frac{W_{i}+W_{i+1}}{2}$$

Step 2: A filter bank, consisting of a low-pass filter and N – 1 bandpass filters, is applied to the limits defined in the frequency spectrum. The Littlewood-Paley and Meyer wavelet structures are used for these functions [26,27].

The empirical scaling function (*ϕ_n_* (ω)) is calculated using the formula in Equation (2).

(2) $$\phi_{n}(\omega )=\left\{\begin{array}{c} 1,\;\;\left|w\right|\leq \left(1-\gamma \right)\omega_{n}\\ \cos\left(\frac{\pi }{2}\beta \left(\gamma ,\omega_{n}\right)\right),\;\;\;\left(1-\gamma \right)\omega_{n}<\left|w\right|\leq \left(1+\gamma \right)\omega_{n}\\ 0,\;\;\;\;\;\;\;\;\;\;\;\;\;\;\;\;\;\;\;\;otherwise \end{array}\right.$$

The empirical wave function (*Ψ_n_* (ω)) is calculated using the formula in Equation (3).

(3) $$\Psi_{n}(\omega )=\left\{\begin{array}{c} \;\;\;\;\;\;\;\;\;\;\;\;\;\;\;\;\;\;\;\;\;\;\;\;\;\;\;\;\;\;\;\;\;1,\;\;\;\;\;\left(1+\gamma \right)\omega_{n}<\left|w\right|<\left(1-\gamma \right)\omega_{n+1}\\ \cos\left(\frac{\pi }{2}\beta \left(\gamma ,\omega_{n+1}\right)\right),\;\;\;\left(1-\gamma \right)\omega_{n+1}\leq \left|w\right|\leq \left(1+\gamma \right)\omega_{n+1}\\ \sin\left(\frac{\pi }{2}\beta \left(\gamma ,\omega_{n}\right)\right),\;\;\left(1-\gamma \right)\omega_{n}\leq \left|w\right|\leq \left(1+\gamma \right)\omega_{n}\\ \;\;0,\;\;\;\;\;\;\;\;\;\;\;\;\;\;\;\;\;\;otherwise \end{array}\right.$$

(4) $$Where\;\alpha \left(\gamma ,\omega_{n}\right)=\beta \left(\left(\frac{1}{2\gamma \omega_{n}}\right)\left(\left|w\right|-\left(1-\gamma \right)\omega_{n}\right)\right)$$

(5) $$\gamma <{min}_{i}\left(\frac{\omega_{n+1}-\omega_{n}}{\omega_{n+1}+\omega_{n}}\right)$$

(6) $$\beta \left(x\right)=\left\{\begin{array}{c} 0,\;\;x\leq 0\\ 1,\;\;x\geq 1\\ \beta \left(x\right)+\beta \left(1-x\right)=1,\;\;x\in (0,1) \end{array}\right.$$

Step 3: The approximate coefficients are calculated using the scaling function, while the detail coefficients are calculated using the wavelet function, as shown in Equations (7) and (8). As a result of the decomposition, an Empirical Mode Function (IMF) is obtained for each band component. The signal is reconstructed as the sum of these modes using the formula in Equation (9). *W_f_*(0, *t*) represents the approximate coefficients, while *W_f_*(*i*, *t*) represents the detail coefficients [26,27].

(7) $$W_{f}\left(0,t\right)=\langle f,\phi_{i}\rangle =\int f\left(\tau \right)\bar{\phi_{i}\left(\tau -t\right)d\tau }$$

(8) $$W_{f}\left(i,t\right)=\langle f,\Psi_{i}\rangle =\int f\left(\tau \right)\bar{\Psi_{i}\left(\tau -t\right)d\tau }$$

(9) $$f\left(t\right)={W_{f}\left(0,t\right)}^{*}\phi_{i}\left(t\right)+\sum_{i=1}^{N}{W_{f}\left(i,t\right)}^{*}\Psi_{i}\left(t\right)$$

### 3.4. Empirical Mode Decomposition (EMD)

Empirical Mode Decomposition is a decomposition method developed by Huang et al. in 1998 [28] for the analysis of nonlinear and non-stationary time series signals. In this method, the signal does not need any predefined basis functions. The signal is decomposed according to its internal structure.

In the EMD method, the input time series is expressed as a sum of a residual term and a varying number of IMFs. In the EMD method, a signal *s*(*t*) is decomposed into a finite number of Intrinsic Mode Functions (IMFs) and a residual term.

(10) $$s\left(t\right)=\sum_{m=1}^{M}c_{m\;}(t)+r_{M}(t)$$

In Equation (10), *c_m_* (*t*) represents the mth IMF, while *r_M_* (*t*) represents the low-frequency residual component of the signal. M denotes the total number of components.

One of the defining criteria for an IMF is that the number of local maxima and minima must be equal to the number of zero-crossings, or differ by no more than one. Furthermore, the mean value of the upper and lower envelopes, defined by the local maxima and minima, must be zero or close to zero at every point. These conditions ensure that the resulting components exhibit narrow-band, meaningful oscillatory structures [29].

Firstly, the local maxima and minima of the signal are determined during the decomposition process. Upper and lower envelopes are created using these points, and a reference signal is obtained by taking the average of these envelopes. Then, this reference signal is subtracted from the original signal. This process is repeated iteratively, and the resulting component is extracted as an IMF once it satisfies the specified criteria. Subsequently, the same process is repeated on the remaining signal to extract all subsequent components [30].

The extracted components represent the signal’s behavior across different time scales. Typically, the initial components contain high-frequency oscillations, whereas the subsequent components reflect slower variations in the signal. This method allows for a more detailed examination of the changes in the signal over time.

### 3.5. Feature Extraction and Selection

EEG signals have a stochastic and non-stationary structure; therefore, the features were extracted from the time, frequency, and nonlinear domains to obtain meaningful information. In this study, three intrinsic mode functions (IMFs) were obtained for each channel from the EEG signals decomposed using EWT and EMD. The rationale for selecting three IMF components in this study is based on the complete representation of physiological frequencies in resting-state EEG signals in the MODMA dataset, maintaining computational efficiency, and avoiding the risk of overlearning. Since a bandpass filter in the 0.5–45 Hz range was applied to denoise the EEG signals, it was decided that the first three IMFs were sufficient to detect the fundamental oscillations associated with MDD (gamma and beta in IMF1, alpha and theta in IMF2, delta in IMF3). Our preliminary experimental evaluations showed that removing higher-order IMF components represents low-frequency fluctuations below 0.5 Hz or computationally generated noise. Furthermore, since 23 different features were removed from each IMF component, having more than three IMFs would increase the size of the feature matrix. This increase in features can lead to the appearance of redundant features that repeat each other, thus carrying the potential for overlearning in machine learning models. For these reasons, limiting the analysis to the first three IMF components is considered to offer the most ideal balance between quality feature representation and classification performance. The features extracted from each IMF component were presented in Table 2. In addition to the extracted features, asymmetry features were also integrated into the analysis to represent functional differences between the left and right brain hemispheres. Differential Asymmetry (DASM) is the arithmetic difference between the same feature values extracted from the symmetric channels of the brain’s two hemispheres [31,32]. Thus, it quantifies neural irregularity and spectral variability. Asymmetry features were calculated by taking the difference between the same feature values obtained from symmetric channel pairs for each IMF component. For example, differential asymmetry values were calculated for the Shannon Entropy (SE) values extracted from the left frontal (F3) and right frontal (F4) channels for each IMF. These hemispheric asymmetry features extracted from each IMF component had a significant contribution to the diagnosis of MDD.

**Figure 2:**
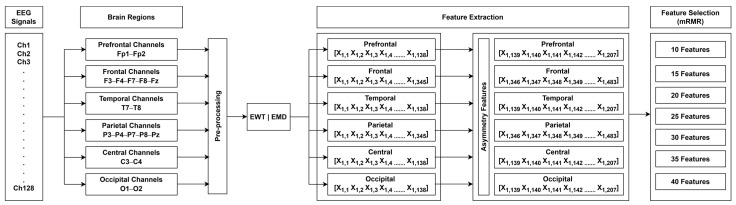
Flowchart of the proposed feature extraction and selection process.

Figure 2 shows the stages of feature extraction, with the prefrontal, frontal, central, parietal, temporal, and occipital brain regions forming the base of the total feature space. In total, 23 features (statistical, Hjorth, entropy, spectral, and AR coefficients) were extracted from each IMF subband for each channel representing regional brain activity. Since these features were extracted from the three IMF components, 69 (23 × 3) features were obtained per channel. In this study, 345 features were extracted from the frontal and parietal regions using 5 channels. When we also integrated the asymmetry features, a total of 483 features were extracted for each segment. In the other regions, 138 features were extracted using 2 channels. When asymmetry features were also integrated, a total of 297 features were extracted for each segment. The extracted features were organized into a matrix on a subject-by-subject basis, with columns representing the features and rows representing the segments in the subjects’ EEG signals. Since these extracted features could increase the risk of overfitting in classification, the mRMR (Minimum Redundancy Maximum Relevance) method was applied as the feature selection method. Notably, mRMR was proposed by Peng and colleagues in 2005 as a filter-based feature selection method [45]. Due to the multi-channel structure of EEG signals and the feature extraction by decomposing them into three IMF components, the mRMR algorithm is more advantages than other methods. Hence, mRMR was chosen as the feature selection method because of some limitations of the others. For example, PCA (Principal Component Analysis) was not used in this study because it risks losing the physiological meaning of features extracted from IMF components and could transform the data into meaningless components. The LASSO method, which only considers the relationships between linear features, eliminates interconnected features that are important for classification. On the other hand, the RFE (Recursive Feature Elimination) method performs extremely slowly on high-dimensional feature matrices. Therefore, it was not preferred to use it in this study to avoid a high computational load and overfitting. Finally, since the Relief and standard MI (Mutual Information) methods focus on features individually, these methods cannot identify and eliminate redundant variables among redundant features—which are derived from IMFs and share the same information content. In conclusion, while mRMR selects the features with the maximum correlation to the target class, it eliminates features that are information redundant with each other. Thus, the classification model is fed with the feature set that has the highest informational value. The mRMR method retains discriminative features while simultaneously reducing the model’s computational load [46]. In this method, features that show a high correlation in MDD and healthy classifications were prioritized. Similar features that were unnecessary in the classification were eliminated. Maximum relevance was calculated using the F-statistic to determine the strong relationship between features in the MDD and HC classes, as shown in Equation (11). Minimum Redundancy was calculated for the MDD and HC classes using the Pearson correlation coefficient, as shown in Equation (12). If these features provide the same information, they create a risk of overfitting in the classification. This method was used to eliminate similar features. The combination formula for ranking in the feature space using the mRMR method was shown in Equation (13) [47,48].

(11) $$maxRL_{f},RL_{f}=\frac{1}{\left|S\right|}\sum_{X_{i}\in S}F\left(X_{i},y\right)\;$$

(12) $$minRD_{p},\;RD_{p}=\frac{1}{{\left|S\right|}^{2}}\sum_{X_{i}\in S}\rho \left(X_{i},X_{j}\right)$$

(13) $$f^{FCQ}\left(X_{i}\right)=\underset{X_{i}\in {Ω}_{S}}{\max}\left\{\frac{F\left(X_{i},y\right)}{\frac{1}{\left|S\right|}\sum_{X_{j}\in S}\left|\rho \left(X_{i},X_{j}\right)\right|}\right\}$$

In this equation, *y* denotes the target class, |*S*| denotes the size of the feature set, and *S* = {*X*1, *X*2, *X*3, ..., *X*n} denotes the feature set. *ρ*(*X_i_*, *X_j_*) is the Pearson correlation between features, and *F*(*X_i_*, *y*) is the F-statistic. According to the mRMR method, the feature importance is *Xi* (*i* ∈ {1, 2, ..., *m*}); *m* is the total number of features, and *f^FCQ^* is the subset in which the selected features are ranked based on their importance scores. The features with the highest importance scores are determined using the mRMR method and ranked in this subset [47,48]. To prevent data leakage, instead of applying mRMR to the entirety of the dataset at once, a subject-based cross-validation method was performed during the feature selection process. Since the validation method employed in this study was subject-based, the mRMR method was applied exclusively to the training data of the N – 1 subjects in each iteration to determine the optimal features. The test data were excluded from this feature selection process.

### 3.6. Classification

Four basic machine learning models—SVM, RUSBoost, Random Forest, and k-NN— were used to classify subjects with major depressive disorder (MDD) and healthy subjects. Additionally, a Meta-Ensemble Model was applied to combine the predictions of these models. The Leave-One-Subject-Out (LOSO) validation method was used during the classification process to prevent data leakage and avoid overfitting. In the LOSO validation method, all EEG segments belonging to a single subject were separated in each test iteration. To prevent data leakage from the EEG segments of the remaining subjects, the steps of mRMR feature selection, model training, and decision threshold optimization for the classification models were performed on the training data of each fold within the LOSO validation cycle. The subjects set aside for the test data were not included in the feature selection processes; the classification models encountered these subjects for the first time during the testing phase. Therefore, the classification performance of the model was measured using data from subjects it had never seen before. To prevent the hyperparameters of the classification models from over-optimizing for a specific subject profile, they were set a priori based on mathematical model stability and clinical diagnostic accuracy. In the SVM model, a Radial Basis Function (RBF) kernel was used with the kernel parameter (ϒ) set to the default scaling and the regularization parameter (C = 1) to detect nonlinear patterns and maximize the margin between classes in the feature space [49]. In the Random Forest model, 100 decision trees were selected to ensure asymptotic stability, and classification was performed using the Bagging architecture. Each tree was trained on a different subset of the data, and no maximum depth limit or pruning was applied to the trees. Thus, the aim was for the trees in the model to capture complex features without being constrained by the high-dimensional feature space [50]. In the k-NN model, the Euclidean distance was used as the distance metric, and the number of neighbors was set to 5. Euclidean distance was chosen to prevent potential outliers in the data from distorting the decision boundaries. Additionally, k = 5 was selected because it represents the most stable balance point between overfitting and underfitting in the binary classification process [51]. In the RUSBoost model, an architecture integrated with Random Under-Sampling was utilized to mitigate class imbalance, training the model with 100 decision trees. This model was used to iteratively minimize classification errors without distorting the original data structure and to increase sensitivity to the MDD class [52]. The Meta-Ensemble model combined the average probabilities of the outputs from these four models to determine the final classification decision [53]. The decision threshold was set to 0.45 based on the sensitivity toward the MDD class, using only the training data. Since the dataset contained 29 healthy controls compared to 24 MDD subjects, methods such as SMOTE that generate synthetic data from external sources were not used to reduce class imbalance. This is because generating synthetic EEG data risks distorting their neurophysiological meaning. Consequently, the a priori fixation of parameters means that the classification accuracy rates obtained in this study stem from the proposed EWT/EMD-based asymmetry features and the mRMR feature selection strategy.

### 3.7. Statistical Analysis

To evaluate whether the observed performance differences between the investigated methods were statistically significant, the Wilcoxon signed-rank test was employed. This non-parametric test was selected because the classification accuracies obtained from different decomposition methods and classifiers represent paired observations and may not satisfy the normality assumption required by parametric statistical tests. A global Wilcoxon signed-rank test was performed to compare the overall classification performances of the EMD- and EWT-based approaches using all classification accuracies obtained across the evaluated brain regions and feature numbers. In addition, pairwise Wilcoxon signed-rank tests were conducted to compare the Random Forest classifier with the other evaluated classifiers. To account for multiple pairwise comparisons, the Bonferroni correction was applied. Statistical significance was determined at a threshold of *p* < 0.05 [46].

### 3.8. SHAP (SHapley Additive exPlanations)

SHAP analysis is an explainable artificial intelligence technique that interprets the predictions of machine learning models by quantifying the contribution of each individual feature to the classification outcome. In this study, SHAP was employed to explain the model’s predictions by identifying how each feature influenced the discrimination between the MDD and HC groups. The contribution of each feature is quantified using the Shapley value, which is calculated according to Equation (14):

(14) $$\phi_{i}=\sum_{S\subseteq F\setminus \left\{i\right\}}\frac{\left|S\right|!\left(\left|F\right|-\left|S\right|-1\right)!}{\left|F\right|!}\left[f\left(S\cup \left\{i\right\}\right)-f\left(S\right)\right]$$

In this equation, *F* denotes the complete set of features, *i* represents the feature under evaluation, and *S* signifies a subset of *F* that excludes feature *i*. *F* acts as a value function to evaluate the performance of a given subset. Thus, the marginal contribution of each examined feature *i* across the entire feature space *F* is determined as $\phi_{i}$. The Shapley value is then computed for a trained classifier *f* and a specific subset S⊆F that isolates feature *i* from the feature space [54].

In the proposed method, the SHAP analysis is integrated directly into the LOSO cross-validation loop to rigorously prevent inter-subject data leakage. During the classification stage, the marginal contribution of each feature to the final decision is computed using a data matrix from test subjects that the Random Forest model has never encountered before. These calculated Shapley values mathematically clarify how the features previously selected by mRMR shape the decision boundaries of the Random Forest classifier. To ensure transparency, the resulting Shapley weights are mapped onto specific electrode channels and anatomical brain regions. Through the global SHAP plot, the relative contributions of features extracted from EEG channels and their corresponding EMD frequency components (IMFs) are explicitly illustrated. Furthermore, the impact of inter-hemispheric asymmetry features within the frontal and prefrontal brain regions on the final MDD or HC decisions is clearly demonstrated. Consequently, the global SHAP visualization successfully translates a complex signal processing and artificial intelligence architecture into a transparent, forward-looking clinical decision-support system by demystifying every step of the MDD diagnostic prediction.

## 4. Results and Discussion

In this study, empirical wavelet transform (EWT) and empirical mode decomposition (EMD) methods were applied to 128-channel EEG signals recorded at rest from the MODMA dataset for the detection of major depressive disorder (MDD). EWT and EMD signal processing methods were applied separately to EEG signals from all cortical regions and separated into 3 IMFs. The MRMR method was applied to the features extracted from each IMF, and feature selection was performed with varying numbers of features. Thereafter, performance results were compared across 5 different classification methods (Support Vector Machine, RUSBoost, Random Forest, k-NN, Meta-Ensemble). The highest accuracy rates, the brain regions where this accuracy was detected, the number of features, and the classification models are presented in Table 3. Leave-One-Subject-Out (LOSO) cross-validation was applied to all classification methods to objectively evaluate across subjects. In the LOSO validation method, the data for one subject was completely separated as the test set at each step. The Meta-Ensemble and Random Forest classification methods were more successful than other classification methods. Although there were anatomical and physiological differences among the 53 subjects in the dataset, major depressive disorder was diagnosed with high accuracy using the EWT- and EMD-based feature space. In particularly, when the EMD-based signal processing method and the Random Forest classification method were used, major depressive disorder was detected with a 98.11% accuracy rate within a [94–100] confidence interval for feature numbers of 15 or more. Additionally, [94–100] confidence intervals (CI) were obtained using a combination of EMD and Random Forest classification methods. These results showed that the variance was low and consistent across the 53 subjects. The model yields consistent and accurate results for the dataset used in this study, though larger external studies are needed to evaluate its true predictive performance. In the study, a high accuracy rate was achieved in the prefrontal and frontal regions. This result supported the frontal asymmetry studies in the literature [55,56,57] regarding the detection of major depressive disorder.

EMD detected nonlinear changes in complex EEG signals more effectively than EWT because it was based on intrinsic mode functions (IMF) that conformed to the local characteristics of the signal. EWT has demonstrated more balanced performance, particularly with a low number of features. However, EWT did not achieve a value as high as EMD’s as the number of features increased. These results indicate that EWT’s fixed filter bank structure has missed details compared to EMD’s adaptive structure. Table 3 shows that the highest performance scores were generally observed in the Prefrontal (PF) and Frontal (F) regions. In particular, the accuracy rates of 98.11% obtained for Random Forest and Meta-Ensemble belong to these two regions. These findings indicate that the major distinguishing features of cognitive activity or neurological status were concentrated in the frontal lobes of the brain. In the EWT method, the Meta-Ensemble model achieved an accuracy rate of 90.57% using 10 features in the Central (C) region, while the RUSBoost model achieved the same accuracy rate using 25 features. The EMD-based RUSBoost model achieved an accuracy rate of 92.45% with 10 features in the Occipital (O) region. However, when the number of features was increased in this region, the accuracy of the RUSBosst algorithm decreased in classifying major depressive disorder. Meta-Ensemble model showed similar performance to the Random Forest classifier. Ensemble learning strategies demonstrated high prediction stability in EEG signal processing. In the prefrontal (PF) region, the EWT-based Random Forest model achieved an accuracy of 92.45% with 20–40 features. The Temporal (T) and Occipital (O) regions achieved an accuracy of 94.34% in the EMD-based Meta-Ensemble model using 10 features. However, when the number of features increased, the performance of these regions was not as stable as that of the frontal region. When the EMD-based RUSBoost algorithm was used, the accuracy rate decreased to 64.15% in the Parietal (P) and Temporal (T) regions. This indicates that the algorithm is not sensitive to the characteristics of these regions. The EWT-based Random Forest method demonstrated an accuracy rate of 94.34% with a selection of 10 features. It was observed that as the number of features increased, the EMD method exhibited a more stable and higher performance curve. The EMD method’s ability to detect changes in nonlinear and non-stationary signals was more distinctive in high-dimensional feature sets. The EMD method’s ability to detect changes in nonlinear and non-stationary signals was more distinctive in high-dimensional feature sets. The detailed performance metrics obtained using different feature numbers and classifiers are summarized in Table 4.

**Figure 3:**
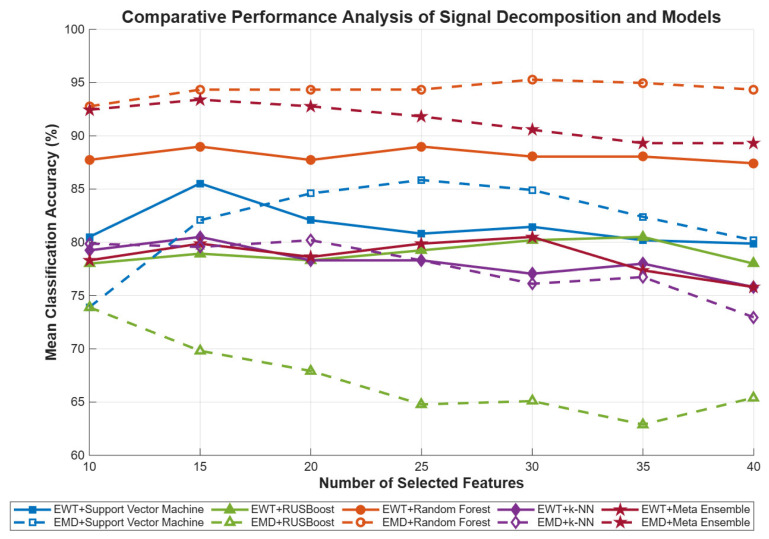
Analysis of Average Model Performance According to Number of Features.

Table 4 presents the performance metrics of the top-performing configurations across different classifiers. The F1-score, ranging from 94.12% to 97.96%, indicates that the distribution across classes was successful. These models are capable of distinguishing both the positive and negative classes. The accuracy rate has been supported with high sensitivity due to the integration of asymmetry features. In a clinical decision-support system, the value of this metric is important because it would be risky to label actual patients as healthy. The EWT-based Random Forest classifier determined an accuracy rate of 94.34% with a selection of 10 features. This result was advantageous in terms of low computational load. Additionally, it demonstrated the potential of EWT for mobile EEG applications. In the parietal region, the EMD-based Random Forest configuration achieved 98.11% accuracy with 20 features. This result indicated that major depressive disorder was also associated with distinctive neurological signals in the posterior regions of the brain. The prefrontal and frontal regions showed consistent performance between 10 and 40 features. These regions have demonstrated more stable performance compared to other regions. The overall classification performance obtained for different feature numbers and classification algorithms is summarized in Figure 3.

Figure 3 presents the average accuracy rates obtained from all cortical regions using LOSO validation on 53 subjects. This graph shows a comparison of the average success rates in five different classification models as signal decomposition methods and the number of features. These analysis results were important for assessing the stability of our study’s methodology and the risk of overfitting. The EMD-Random Forest combination demonstrated the highest and most consistent performance across all feature selections. This combination achieved the highest average accuracy of 95.28% with the selection of 30 features.

Furthermore, it demonstrated excellent stability with an accuracy ranging from 94% to 95% across 15 to 40 features. The EMD-Meta Ensemble combination demonstrated strong performance, with an accuracy rate of 93.40% using 15 features. However, the accuracy rate for this combination decreased as the number of features increased, dropping to 89%. The most significant decline was observed in the EMD-RUSBoost combination. It achieved an accuracy rate of 73.90% with 10 features, which dropped to 62.89% as the number of features increased. The EMD-SVM combination exhibited a downward trend after attaining a peak accuracy of 85.85% with 25 features. In the EMD-kNN combination, an accuracy rate of 80.19% with 20 features decreased to 72.96% as the number of features increased. The EWT-SVM combination demonstrated the highest performance, attaining an accuracy rate of 85.53% with 15 features. However, when the number of features increased, the accuracy rate decreased to 80%. In the EWT-RUSBoost combination, when the number of features was increased from 10 to 35, the accuracy improved from 77.99% to 80.50%. In the EWT-Random Forest combination, an accuracy rate of 87–88% was achieved across all feature selections, and the graph was stable. The EWT-kNN combination, which achieved the best accuracy rate of 80.50% with 15 features, decreased to 75.79% with 40 features. These results indicate that distance-based classifiers exhibit a performance decline when selecting high-dimensional features. When 30 features were selected, the EWT-Meta Ensemble combination achieved the highest success rate of 80.50%. However, the accuracy rate decreased to 75% after selecting 35 features. These results demonstrate that both Random Forest and Meta-Ensemble classifiers achieve an accuracy of over 90% using the EMD method across 53 participants in the LOSO validation. This supports the notion that ensemble methods should be preferred over classifiers such as SVM and k-NN for stochastic signals like EEG.

**Figure 4:**
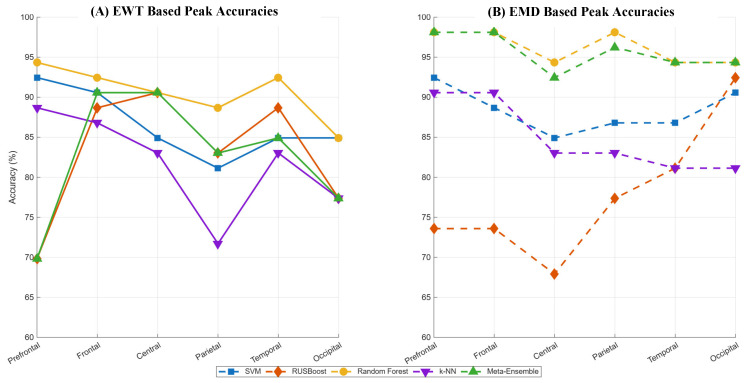
Comparative analysis of the classification performance of the EWT and EMD methods for different brain regions.

Figure 4 presents a comparative analysis of the peak classification accuracies obtained using EWT and EMD methods across feature sets ranging from 10 to 40 for each brain region.

As shown in Figure 4, the effect of signal decomposition methods varies across brain regions. In the EWT method, the highest performance was achieved in the prefrontal region using Random Forest and SVM models. However, all classification models experienced a decline in accuracy within the parietal region. Although the Meta-Ensemble and RUSBoost models achieved a 70% accuracy rate in the prefrontal region, superior accuracy rates were observed in other brain regions. The EWT-RUSBoost combination achieved its lowest accuracy rate of 70% in the prefrontal region. It is noteworthy that the accuracy rate increased in the frontal and central regions, reaching the 90% range. The features obtained from the EWT-based filter bank were not distinctive for the RUSBoost algorithm in the prefrontal region. When the EMD method was evaluated, it demonstrated greater robustness and higher accuracy compared to EWT. The Random Forest and Meta-Ensemble models achieved optimal performance in the Prefrontal and Frontal regions, with an accuracy of 98.11%. In the EMD graph, the RF and Meta-Ensemble models exhibited highly comparable performance profiles. RUSBoost underperformed relative to all other models, with the exception of its performance in the Occipital region. Features extracted from EMD were not classified in the RUSBoost structure, and there was a decline in performance, particularly in the Central region. The accuracy rate decreased in the transition from the prefrontal to the frontal region in the EWT graph, whereas this performance was maintained, except for the SVM model, in the EMD graph. Features extracted from the electrical activity in the prefrontal and frontal regions of the brain using the EMD signal processing method were more discriminative for the diagnosis of MDD. In the regional peak accuracy analysis presented in Figure 4, EMD-based feature extraction demonstrates higher accuracy and more consistent performance across all brain regions compared to the EWT method. These findings, obtained through LOSO validation on 53 subjects, indicated that the EMD-Random Forest combination provides the most robust feature space despite inter-subject anatomical and physiological differences. These results demonstrated that EMD’s decomposition of the signal was more effective for MDD diagnosis.

The EMD-based configurations generally achieved higher classification performances than the EWT-based configurations. To statistically evaluate whether the observed performance differences were significant, a global Wilcoxon signed-rank test was performed using all classification accuracies obtained across the evaluated classifiers, brain regions, and feature numbers. The EMD-based approach achieved a higher overall mean classification accuracy (82.50%) than the EWT-based approach (81.08%). The global Wilcoxon signed-rank test indicated that this difference was statistically significant (*p* = 0.0206), suggesting a modest but significant overall performance advantage of EMD over EWT. Furthermore, Table 5 presents the statistical comparison between the Random Forest classifier and the other evaluated classifiers using Wilcoxon signed-rank tests with Bonferroni correction. The results demonstrated that Random Forest significantly outperformed all alternative classifiers for both EMD- and EWT-based feature sets (all Bonferroni-adjusted *p*-values < 0.001), supporting the robustness and superior classification capability of the Random Forest classifier across the evaluated experimental configurations.

**Figure 5:**
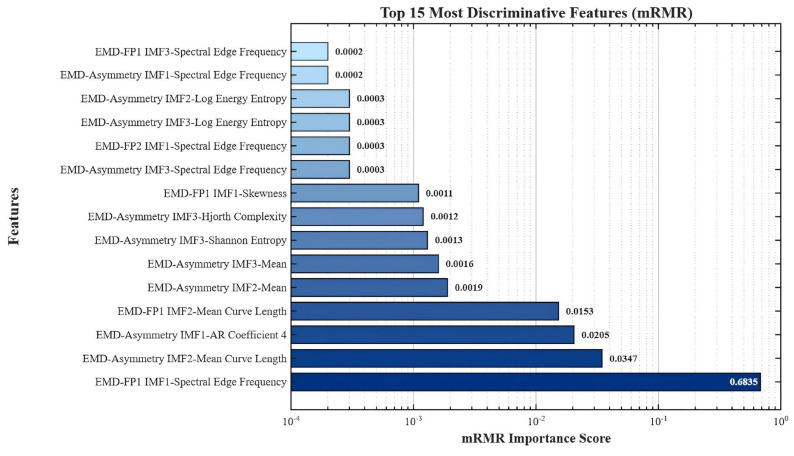
The mRMR importance scores for EMD-based features of the prefrontal region.

**Figure 6:**
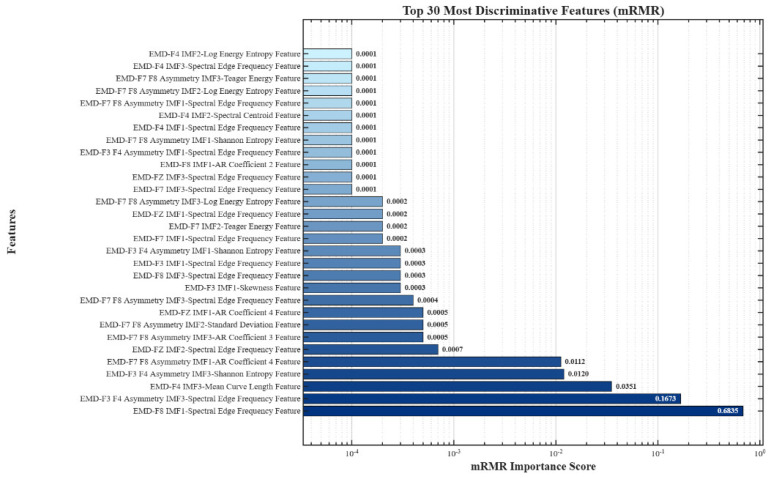
The mRMR importance scores for EMD-based features in the frontal region.

Figure 5 and Figure 6 present the top 15 features in the prefrontal region and the top 30 features in the frontal region identified by the mRMR algorithm, along with their importance scores.

Figure 5 and Figure 6 present the top 15 features in the prefrontal region and the top 30 features in the frontal region identified by the mRMR algorithm, along with their importance scores. The Spectral Edge Frequency (SEF) feature, extracted from the first internal mode function (IMF1) decomposed with EMD, stands out in both regions. This feature contributed to classification success with an importance score of 0.6835. In the prefrontal region, the Mean Curve Length from IMF2, the AR Coefficient from IMF1, the Mean values from IMF2 and IMF3, Shannon Entropy and Hjorth complexity from IMF3, and asymmetry features from the Fp1−Fp2 channels significantly contributed to the classification success of the models. The Mean Curve Length from IMF2 and Skewness from IMF1 in the Fp1 channel were also effective for classification when integrated with asymmetry-based features. In the frontal region, the asymmetry Spectral Edge Frequency and Shannon entropy features were derived from IMF3 in the F3–F4 channels; the asymmetry AR Coefficient features were derived from IMF1 and IMF3 in the F7–F8 channels; asymmetry standard deviation features from IMF2 in the F7–F8 channels were determined using the mRMR algorithm and improved the models’ classification performance. The Mean Curve Length from IMF3 in the F4 channel and the Spectral Edge Frequency from IMF2 in the Fz channel were also features that improved classification performance. Figure 5 and Figure 6 demonstrated that asymmetry features, particularly the electrical difference between the two brain hemispheres, were significant in classification.

The classification performance was particularly influenced by the asymmetry features when examining the mRMR importance scores. Spectral Edge Frequency was identified as the most influential feature. The asymmetry-focused feature selection in this study improved the model’s subject-independent performance.

**Figure 7:**
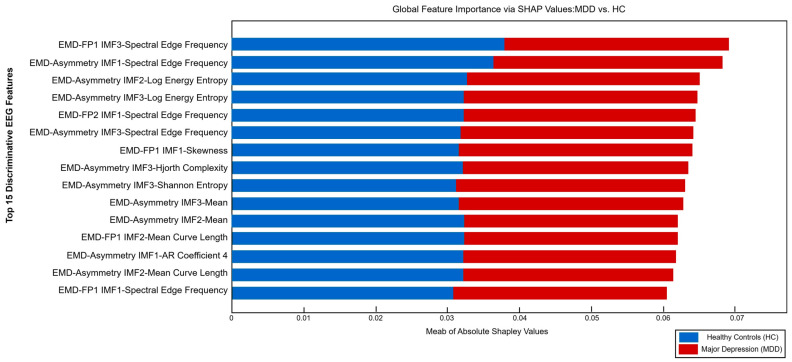
SHAP global feature importance analysis for the top 15 discriminative EEG features in the prefrontal region.

**Figure 8:**
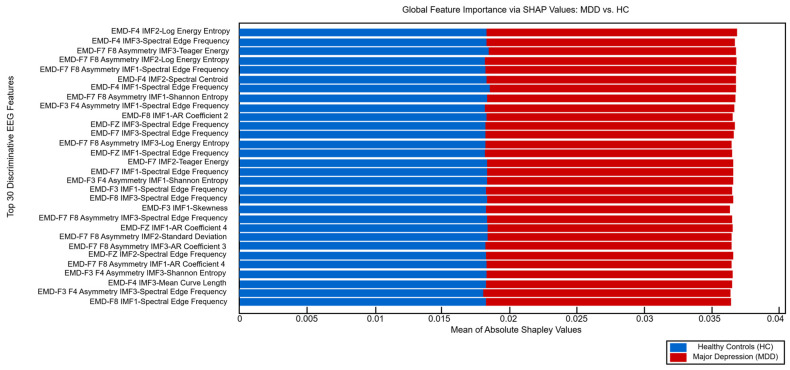
SHAP global feature importance analysis for the top 30 discriminative EEG features in the frontal region.

Figure 7 and Figure 8 present the SHAP (Shapley Additive Explanations) summary plots, illustrating the contribution of features extracted from the prefrontal and frontal regions to the models’ predictive accuracy.

The mRMR feature selection identified which features are the most discriminative for classification performance. Figure 7 and Figure 8 present the Global SHAP (Shapley Additive Explanations) summary plots, generated to evaluate generalized feature contributions across LOSO-validated models. To prevent data leakage and overfitting, the Global SHAP values were obtained directly from the models within the LOSO validation loop, rather than from a single model trained on the entire dataset. These plots depict the mean absolute Shapley values, illustrating the contributions of the extracted features to correct predictions for the major depressive disorder class (represented in red) and the healthy control class (represented in blue). Figure 8 presents the global SHAP plot for the frontal region, indicating that the contributions of the features to the MDD and Healthy classes are nearly balanced. The selected features were nearly equally useful in distinguishing between the two classes. Since high correlations among multi-channel EEG features can mislead SHAP interpretations, this validation strategy was applied. The selected features were nearly equally informative in distinguishing between the two classes. Since high correlations among multi-channel EEG features can skew SHAP interpretations, this validation strategy was applied. Highly correlated and redundant features were filtered out using the mRMR algorithm prior to model training. Subsequently, the feature importance trends derived from Global SHAP were cross-checked across the subjects within the LOSO loop. Subsequently, the feature importance trends derived from Global SHAP were cross-checked using this independent mRMR algorithm. Similar to the mRMR feature selection results, the global SHAP plots also confirmed that the asymmetry features extracted via EMD significantly contributed to the models’ classification performance. According to the global SHAP plot for the prefrontal region shown in Figure 7, the Spectral Edge Frequency of IMF3 and the Asymmetry Spectral Edge Frequency of IMF1—both derived from the Fp1 channel—have the most pronounced and consistent effect on the model’s class decision (with a mean absolute SHAP value of approximately 0.07), outperforming the remaining features.

**Figure 9:**
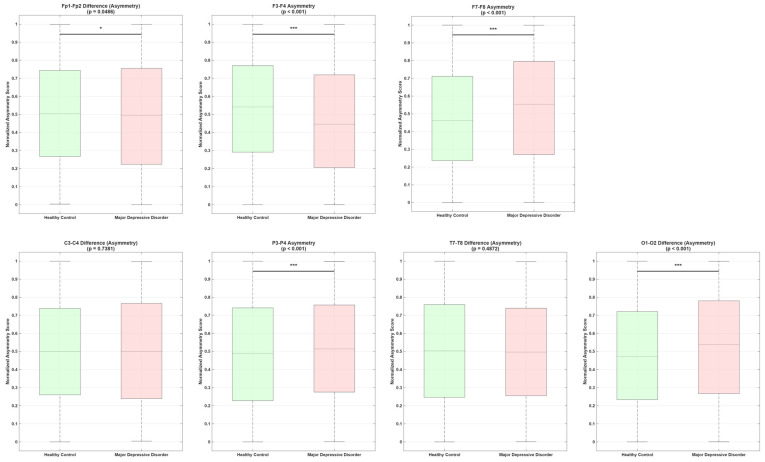
Normalized hemispheric asymmetry scores for HC and MDD classes across different brain regions. Significance levels: * *p* < 0.05 indicates statistically significant difference; *** *p* < 0.001 indicates highly statistically significant difference.

Figure 9 presents the normalized asymmetry scores calculated for the HC and MDD classes, based on hemispheric asymmetry across various brain regions. In accordance with statistical guidelines, the asymmetry scores obtained from each brain region and channel pair were subjected to the Lilliefors test to evaluate whether the data followed a normal distribution [58]. If the test results demonstrated a normal distribution, the Independent Samples *t*-Test was employed to compare the MDD and HC groups [59]. Conversely, if the data deviated from a normal distribution, the Mann–Whitney U test—a non-parametric alternative—was applied [60]. As a result of these analyses, *p*-values between the classes were calculated. A *p*-value of less than 0.05 (*p* < 0.05) indicated that the observed difference between the MDD and HC groups was statistically significant, confirming it did not arise from random chance. Brainwave signals exhibit significant inter-subject variability. Furthermore, evaluating the overall dataset using a single statistical test can lead to misleading results. For this reason, the use of two different tests—the *t*-test and the Mann–Whitney U test—was established to select the appropriate statistical method according to the distribution structure of each feature. The statistical analyses were performed on the hemispheric asymmetry features calculated from the relationships between channel pairs in the left and right brain hemispheres. The box plots indicated statistical significance tests. These graphs statistically reinforced the prioritization of asymmetry features and the model’s achievement of an accuracy rate of 98.11% in the prefrontal/frontal regions. The graphs show that brain activity associated with major depressive disorder was concentrated in the frontal lobes. Asymmetry scores in the Fp1–Fp2 channels demonstrated a statistically significant difference between the HC and MDD classes (*p* = 0.0486). Prefrontal asymmetry scores in the MDD class exhibited greater variability compared to the healthy controls class, with the two classes showing a statistically significant difference. The asymmetry scores in the F3–F4 and F7–F8 channel pairs indicated a high degree of differentiation between the HC and MDD classes (*p* < 0.001). Hemispheric activation differences represented a potential candidate marker for frontal lobe functional alterations within the dataset used in this study. The prioritization of the Spectral Edge Frequency and Shannon Entropy asymmetry features in these channels was confirmed to be effective in the classification success of this statistical significance. There was no statistically significant difference in asymmetry scores in the C3–C4 and T7–T8 channel pairs between the HC and MDD classes (*p* = 0.7381 and *p* = 0.4872). It appears that asymmetry features extracted from the central and temporal regions provided lower discriminative value compared to those from the prefrontal and frontal regions. The asymmetry scores in the O1–O2 and P3–P4 channel pairs demonstrated a highly significant difference between the HC and MDD classes (*p* < 0.001). The analysis using LOSO validation yielded a high accuracy rate of 98.11% across the prefrontal, frontal, and parietal regions. The primary factor that contributes to this high accuracy was asymmetry characteristics. Asymmetry features extracted from the Fp1–Fp2, F3–F4, F7–F8, and P3–P4 channel pairs derived from IMFs using the EMD method presented a statistically significant difference between the HC and MDD classes. The hemispheric asymmetry analysis across these channel pairs also statistically confirmed the neurophysiological foundations of this study. In conclusion, the asymmetry features extracted from IMFs obtained using the EMD method have made significant contributions to the model’s classification accuracy. Asymmetry-based measures have been shown to be a promising tool for exploring hemispheric lateralization differences of the brain in major depressive disorder. Asymmetry in the prefrontal and frontal lobes, along with changes in the power spectrum, was identified as an important candidate feature for MDD classification.

### Discussion of Findings and Limitations

Resting-state EEG signals offer promising biomarkers for Major Depressive Disorder (MDD), but the application of these signals in a clinical setting using explainable artificial intelligence (XAI) models remains a fundamental challenge. This study investigated the effectiveness of EWT- and EMD-based signal decomposition methods for the detection of major depressive disorder using resting-state EEG recordings. The results demonstrated that EMD generally provided higher and more stable classification performance than EWT across different numbers of selected features, brain regions, and classification algorithms. The highest classification accuracy (98.11%) was achieved using EMD-based feature extraction in combination with the Random Forest classifier. In addition, the prefrontal and frontal regions consistently produced better classification results than the remaining brain regions, indicating that depression-related EEG alterations appear to be more pronounced in frontal brain regions.

To better position the proposed framework within the existing literature, the obtained results were compared with previous studies conducted using the MODMA dataset. However, direct comparison of reported classification accuracies should be approached with caution because the studies differ considerably in terms of validation strategies and evaluation protocols. While some studies performed classification using segmented EEG epochs and conventional k-fold cross-validation procedures, others adopted subject-level evaluation schemes. Since segmented EEG samples originating from the same participant may appear in both training and testing sets, the resulting performance estimates may not be directly comparable to subject-independent validation protocols. In contrast, leave-one-subject-out (LOSO) cross-validation evaluates the model using entirely unseen subjects and therefore provides a more rigorous assessment of generalization performance. For this reason, Table 1 includes information regarding the validation strategy and evaluation level whenever such information was available.

Table 1 shows that a wide variety of feature extraction and classification approaches have been applied to the MODMA dataset. Wang et al. [14] reported an accuracy of 94.72% using connectivity-based features and a W-GCN-GRU architecture, whereas Liu et al. [15] achieved 98.30% accuracy using graph-based representations of EEG activity. Esmi et al. [19] reported approximately 96% accuracy using a Transformer-based multimodal framework, while Yousufi et al. [16] achieved 97.53% accuracy by combining EEG and speech information. Among studies for which subject-level evaluation was explicitly reported, Taşcı et al. [12] achieved 83.96% accuracy using handcrafted features and a k-nearest-neighbor classifier, whereas Gülenç et al. [18] reported 98.88% accuracy using EWT-based feature extraction and an ensemble learning framework. In the present study, the highest accuracy was 98.11%, obtained using EMD-based feature extraction and the Random Forest classifier. Although the reported performance is comparable to the best results available for the MODMA dataset, the proposed framework additionally provides regional EEG analysis and model interpretability through SHAP-based explanations.

A notable aspect of the proposed approach is the regional analysis of EEG activity. By examining the prefrontal, frontal, central, parietal, temporal, and occipital regions separately, it was possible to evaluate the spatial distribution of depression-related EEG alterations. The results consistently showed that the prefrontal and frontal regions provided the most discriminative information for classification. This finding is consistent with previous studies reporting abnormal frontal activity and altered hemispheric asymmetry in patients with major depressive disorder [55,56,57].

The importance of frontal brain activity was further supported by both the mRMR feature selection results and the SHAP analysis. Features extracted from the prefrontal and frontal regions were repeatedly identified among the most informative variables contributing to classification performance. The agreement between these independent analyses strengthens the evidence that frontal EEG activity contains important information related to depressive symptoms. These findings suggest that depression-related electrophysiological alterations are predominantly reflected in frontal brain networks.

Another advantage of the proposed framework is that the contribution of individual features can be examined directly. This makes it possible to identify which EEG characteristics have the greatest influence on classification performance and facilitates the interpretation of the obtained results. Consequently, the proposed approach provides not only high classification performance but also additional information regarding the regional characteristics of depression-related EEG activity.

Although the EMD-based Random Forest model proposed in this study achieved a high accuracy rate of 98.11%, there are some methodological limitations regarding the method’s clinical applicability. Firstly, this study was performed on a small dataset consisting of 53 participants. Although the LOSO validation strategy was applied to prevent the dependence of the model on the subject, due to the limited sample size, further analysis is required in a larger and more heterogeneous population. Second, the lack of an independent external validation dataset—which could assess the success of the proposed model across different EEG devices and signal recording standards—is another limitation. Third, when applying feature selection methods such as mRMR, data leakage may occur if the validation boundaries are not clearly distinguished. LOSO cycles were controlled to prevent the training and test data from mixing with each other. Fourth, there is a need to use more advanced automatic algorithms to remove eye- and muscle-related artifacts during the signal preprocessing stage. Fifth, the subtypes of major depressive disorder (MDD) (melancholic, atypical, etc.) and symptom intensity levels (mild, moderate, severe) were not included in the analysis. Furthermore, there is currently no monitoring system that tracks changes in asymmetry features throughout different stages of patients’ treatment. Finally, the proposed model has not yet been tested in a clinical setting. To facilitate its integration into a clinical decision-support system, the proposed model requires further validation through real-time evaluation in psychiatric clinical practice.

## 5. Conclusions

In this study, a biological and objective methodology based on the analysis of brain regions using EEG signals is presented for major depressive disorder, which is currently diagnosed by psychiatrists solely based on clinical interviews. The proposed methodology analyzed the non-stationary and complex structure of EEG signals using signal processing methods such as EWT and EMD. Instead of using a single algorithm, the study develops a feature-based decision-support system that consists of EWT/EMD, mRMR feature selection, LOSO validation, and SHAP analysis.

Experimental results indicate that EMD was more successful in analyzing the complex structure of EEG signals for the diagnosis of MDD. In addition, it was observed that changes in brain electrical activity associated with MDD were primarily localized in the prefrontal and frontal regions. In particular, this study demonstrated that features reflecting interhemispheric asymmetry were highly discriminative in the classification of major depressive disorder. Asymmetry in the prefrontal and frontal lobes, along with changes in the power spectrum, was found to be a promising candidate biomarker for MDD diagnosis. This study’s most significant contributions were its interpretability through mRMR importance scores and SHAP analysis, which is based on explainable artificial intelligence. The proposed EMD-based feature space, validated through a LOSO scheme on 53 subjects, achieved a 98.11% classification accuracy in the prefrontal and frontal regions using Random Forest and Meta-Ensemble models. The results of the SHAP analysis demonstrated that the Spectral Edge Frequency in IMF1 was the feature that contributed most significantly to the classification accuracy. This feature was followed by Shannon entropy, AR coefficients, and mean curve length derived from asymmetry.

In conclusion, the study reported which features were effective in distinguishing between the MDD and healthy control classes. It is recommended that this model be tested in larger patient groups and used for antidepressant treatment monitoring in the future. By integrating this model with portable EEG devices, a decision-support system can be developed for early diagnosis and treatment processes in hospitals. This study provides a foundation for clinical decision-support systems that leverage biological markers for the diagnosis of psychiatric patients.

## Figures and Tables

**Table 1:** Comparison of related studies on the detection of major depressive disorder using the MODMA dataset.

Year	Study	Channels	Data	Feature Extraction	Method	Validation	Acc (%)	Ref.
2022	Cai et al.	128 + 3	EEG + Speech	Dataset	-	-	-	[9]
2022	Chang et al.	128	EEG	STFT+HHT+spatial	DL (CNN + BiLSTM)	-	~70	[10]
2023	Ksibi et al.	128	EEG	Statistical+nonlinear features	XGBoost + RF + 1D-CNN	-	~90+	[11]
2023	Taşcı et al.	128	EEG + Speech	DWT + textural (TPTLP) + statistical	ML (KNN + voting)	LOSO	~83.96	[12]
2024	Khan et al.	3	EEG	Temporal + FS	ML (BF-Tree)	Train/Test Split	96.36	[13]
2024	Wang et al.	128	EEG	Connectivity	GCN+GRU	10-fold CV	94.72	[14]
2024	Liu et al.	128	EEG	Graph features	GNN	10-fold CV	98.3	[15]
2024	Yousufi et al.	128	EEG + Speech	STFT + Mel	CNN	-	~95	[16]
2024	Gupta et al.	3	EEG	Statistical	ML	Train/Test Split	~90+	[17]
2024	Gülenç et al.	3	EEG	Time domain, frequency domain, and nonlinear	EWT-Ensemble	10-fold CV	98.88	[18]
2026	Esmi et al.	128	EEG + Speech	Transformer fusion	Transformer	-	~96	[19]

Abreviations: EEG: Electroencephalography; STFT: Short-Time Fourier Transform; HHT: Hilbert–Huang Transform; DWT: Discrete Wavelet Transform; FS: Feature Selection; CNN: Convolutional Neural Network; BiLSTM: Bidirectional Long Short-Term Memory; GCN: Graph Convolutional Network; GRU: Gated Recurrent Unit; GNN: Graph Neural Network; ML: Machine Learning; EWT: Empirical Wavelet Transform.

**Table 2:** Definitions and mathematical expressions of the features extracted.

	Features	Definition	Formula
**Statistical Time Domain Features**	**Skewness**	It measures the symmetry of the signal’s amplitude distribution [33,34].	$Skewness=\frac{\frac{1}{N}\sum_{i=1}^{N}{\left(x_{i}-\bar{x}\right)}^{3}}{\sigma^{3}}$
	**Kurtosis**	It measures the density of peaks in the signal [33,34].	$Kurtosis=\frac{\frac{1}{N}\sum_{i=1}^{N}{\left(x_{i}-\bar{x}\right)}^{4}}{\sigma^{4}}-3$
	**Mean**	It is the arithmetic mean representing the center of the signal [34,35].	$\bar{x}=\frac{1}{N}\sum_{i=1}^{N}x_{i}$
	**Median**	It is the value exactly in the middle when the values of the samples in the signal are ranked [35,36].	If N is odd; $Median=x_{\left(\frac{N+1}{2}\right)}$ If N is even; $Median=\frac{1}{2}\left(x_{\left(\frac{N}{2}\right)}+x_{\left(\frac{N}{2}+1\right)}\right)$
	**Standard Deviation**	It is a measure of how far the signal values deviate from their mean [34,35].	$\sigma =\sqrt{\frac{\sum_{i=1}^{N}{\left(x_{i}-\bar{x}\right)}^{2}}{N}}$
	**Variance**	It measures the average spread of the signal [33,37].	$Var=\frac{1}{N-1}\sum_{i=1}^{N}{\left(x_{i}-\bar{x}\right)}^{2}$
	**Mean Absolute Value (MAV)**	It is the average of the absolute values of the signal [38].	$MAV=\frac{1}{N}\sum_{i\_1}^{N}\left|x_{i}\right|$
**Hjorth Parameters**	**Activity**	It is the total power of the signal [33].	$Activity=Var(x_{i})$
	**Mobility**	It is the average frequency of the signal’s power spectrum [33,35].	$Mobility=\sqrt{\frac{Var(x_{i}^{\prime })}{Var(x_{i})}}$
	**Complexity**	It measures how the signal is sinusoidal [33,35].	$Complexity=\frac{Mobility(x_{i}^{\prime })}{Mobility(x_{i})}$
**Entropy Features**	**Shannon**	It measures the uncertainty and randomness of the signal [33,35].	$Entropy=-\sum_{i=1}^{n}p\left(x_{i}\right){log}_{2}p\left(x_{i}\right)$
	**Log Energy**	It measures the signal’s energy density logarithmically [33].	$E_{log}=\sum_{i}\log\left(p_{i}^{2}\right)$
**Spectral Features**	**Spektral Centroid**	It is the center of gravity of the power spectrum [37,39].	$SC=\frac{\sum_{k=1}^{WfL}kX_{i}(k)}{\sum_{k=1}^{WfL}X_{i}(k)}$
	**Spectral Spread**	It measures how the power of the signal is distributed on the spectrum [37,39].	$SS=\sqrt{\frac{\sum_{k=1}^{WfL}{\left(k-SC\right)}^{2}.\;X_{i}(k)}{\sum_{k=1}^{WfL}X_{i}(k)}}$
	**Spektral Edge Frequency**	It is the frequency value at which the total spectral power is below a certain percentage [40,41].	$\sum_{i=1}^{x}{\left|mag\right|}^{2}=0.95\;x\;\sum_{i=1}^{n}{\left|mag\right|}^{2}\;;\;SEF=freq\left(x\right)\;\;\;\;\;$ (mag= magnitude of FFT coefficients)
**Energy and Waveform Features**	**Mean Curve Length**	It is the total length of the signal’s waveform [42].	$MCL=\frac{1}{N}\sum_{n=1}^{N-1}\left|x_{i+1}-x_{i}\right|$
	**Teager Energy Operator**	It detects the instantaneous changes in both the amplitude and frequency of the signal [40].	$Ѱ\left(x_{i}\right)=x_{i}^{2}-x_{i-1}x_{i+1}$
	**Mean Energy**	It is the mean energy value of the signal [34].	$E_{mean}=\frac{1}{N}\sum_{i=1}^{N}{\left|x_{i}\right|}^{2}$
	**Root Mean Square (RMS)**	It measures the signal’s effective power [38,43].	$RMS=\sqrt{\frac{1}{N}\sum_{i=1}^{N}x_{i}^{2}}$
**Model-Based Features**	**AR (Autoregressive) Coefficients**	It is an autoregressive (AR) model in which the current value is estimated using previous values in the signal [44].	$x\left(n\right)=\sum_{k=1}^{p}a_{k}x\left(n-k\right)+e(n)$

**Table 3:** Classification Performance Across Various Brain Regions and Feature Numbers Using EWT and EMD Methods.

Number of Features	Method	Accuracy [%95 Confidence Interval] (Brain Region)				
		Support Vector Machine	RUSBoost	Random Forest	k-NN	Meta-Ensemble
10	EWT	92.45 [85–99] (PF)	88.68 [80–97] (T/C)	94.34 [88–100] (PF)	88.68 [80–97] (PF)	90.57 [82–98] (C)
	EMD	81.13 [70–91] (C)	92.45 [85–99] (O)	94.34 [88–100] (PF/F/T/O)	84.91 [75–94] (PF)	94.34 [88–100] (T/O)
15	EWT	92.45 [85–99] (PF)	88.68 [80–97] (F/C)	94.34 [88–100] (PF)	86.79 [77–96] (PF)	90.57 [82–98] (F)
	EMD	90.57 [82–98] (PF/O)	77.36 [65–88] (P)	98.11 [94–100] (PF)	90.57 [82–98] (PF)	98.11 [94–100] (PF)
20	EWT	88.68 [80–97] (PF)	88.68 [80–97] (F/C)	92.45 [85–99] (PF)	88.68 [80–97] (PF)	90.57 [82–98] (F)
	EMD	92.45 [85–99] (PF)	75.47 [63–87] (P)	98.11 [94–100] (PF/P)	86.79 [77–96] (PF)	98.11 [94–100] (PF)
25	EWT	88.68 [80–97] (PF)	90.57 [82–98] (C)	92.45 [85–99] (PF/T)	86.79 [77–96] (PF/F)	90.56 [82–98] (F)
	EMD	90.57 [82–98] (PF)	67.92 [55–80] (T/O)	98.11 [94–100] (PF)	83.02 [72–93] (P)	96.23 [91–100] (PF/F)
30	EWT	90.57 [82–98] (F)	88.68 [80–97] (C)	92.45 [85–99] (PF)	86.79 [77–95] (F)	86.79 [77–95] (F/C)
	EMD	92.45 [85–99] (PF)	69.81 [57–82] (O)	98.11 [94–100] (PF/F)	83.02 [72–93] (P)	98.11 [94–100] (PF)
35	EWT	88.67 [80–97] (F)	88.68 [80–97] (F/C/T)	92.45 [85–99] (PF)	88.68 [80–97] (PF)	86.79 [77–96] (C)
	EMD	88.68 [80–97] (PF)	64.15 [51–77] (F/P/T)	98.11 [94–100] (PF/F)	90.57 [82–98] (F)	98.11 [94–100] (F)
40	EWT	86.79 [77–96] (PF/F)	90.57 [82–98] (C)	92.45 [85–99] (PF)	84.91 [75–94] (PF/F)	88.68 [80–97] (C)
	EMD	86.79 [77–96] (PF/P)	69.81 [57–82] (T/O)	98.11 [94–100] (F)	81.13 [70–91] (F)	96.23 [91–100] (F)

PPF: Prefrontal, F: Frontal, T: Temporal, C: Central, P: Parietal, O: Occipital. The values in parentheses represent the brain region where the result was obtained.

**Table 4:** Optimal Classification Achievements and Performance Metrics for EWT and EMD Methods.

Method	Classifier	Brain Region	Number of Features	Accuracy (%) [%95 CI]	Sensitivity (%)	Specificity (%)	Precision (%)	F1-Score (%)
EWT	Random Forest	Prefrontal	10, 15	94.34 [88–100]	100	89.65	88.89	94.12
EMD	Meta-Ensemble	Prefrontal	15, 20, 30	98.11 [94–100]	95.83	100	100	97.87
EMD	Random Forest	Prefrontal	15, 20, 25, 30, 35	98.11 [94–100]	100	96.55	96	97.96
EMD	Random Forest	Frontal	30,35,40	98.11 [94–100]	100	96.55	96	97.96
EMD	Meta-Ensemble	Frontal	35	98.11 [94–100]	100	96.55	100	97.87
EMD	Random Forest	Parietal	20	98.11 [94–100]	100	96.55	96	97.96

**Table 5:** Statistical comparison between Random Forest and the other evaluated classifiers using the Wilcoxon signed-rank test with Bonferroni correction.

Method	Comparison	Mean RF Accuracy (%)	Mean Other Accuracy (%)	*p*-Value	Bonferroni-Adjusted *p*-Value
EMD	Random Forest vs. SVM	94.34	81.98	3.57 × 10^−8^	2.86 × 10^−7^
EMD	Random Forest vs. RUSBoost	94.34	67.11	1.65 × 10^−8^	1.32 × 10^−7^
EMD	Random Forest vs. k-NN	94.34	77.67	1.65 × 10^−8^	1.32 × 10^−7^
EMD	Random Forest vs. Meta-Ensemble	94.34	91.39	1.60 × 10^−5^	1.28 × 10^−4^
EWT	Random Forest vs. SVM	88.13	81.49	6.58 × 10^−7^	5.27 × 10^−6^
EWT	Random Forest vs. RUSBoost	88.13	79.01	6.38 × 10^−7^	5.11 × 10^−6^
EWT	Random Forest vs. k-NN	88.13	78.16	3.57 × 10^−8^	2.86 × 10^−7^
EWT	Random Forest vs. Meta-Ensemble	88.13	78.61	5.16 × 10^−7^	4.13 × 10^−6^

Statistical comparisons were performed using the Wilcoxon signed-rank test. Bonferroni correction was applied to account for multiple pairwise classifier comparisons.

## Data Availability

The EEG data used in this study were recorded at rest using a 128-channel HydroCel Geodesic Sensor Net (HCGSN). Ethical approval was granted by the Local Ethics Committee for Biomedical Research at Lanzhou University Second Hospital, China. The dataset is hosted on the MODMA open-access database https://modma.lzu.edu.cn/data/index/ (accessed on 26 June 2026) and was accessed following the completion of a formal registration and data request form.
